# Supplementary material for: Examining the diagnostic accuracy of artificial intelligence for detecting dental caries across a range of imaging modalities: An umbrella review with meta-analysis
Source: PLoS One. 2025 Aug 13;20(8):e0329986. doi: 10.1371/journal.pone.0329986 (PMC12349118; doi:10.1371/journal.pone.0329986)
Supplement: S1 Table — The “1” implies a checkmark, that is the study is included “0” implies that the study is not included in the review in question. (DOCX) [file pone.0329986.s001.docx]

**S1 Table.** **The citation matrix of primary studies included in the systematic reviews for the use of AI in the detection of dental caries.** The “1” implies a checkmark, that is the study is included “0” implies that the study is not included in the review in question

| **Study** | **Talpur** [1] | **Reyes** [2] | **Moharrami** [3] | **P-Privado** [4] | **M-Rahimi** [5] | **R-León** [6] | **Khanagar** [7] | **Khanagar** [8] | **Khanagar** [9] | **Zanini** [10] | **Ndiaye** [11] | **Ammar** [12] | **Rokhshad** [13] | **Albano** [14] | **Total** |
| --- | --- | --- | --- | --- | --- | --- | --- | --- | --- | --- | --- | --- | --- | --- | --- |
| **Lee et al.**[15] | **1** | **1** | **0** | **1** | **1** | **1** | **1** | **0** | **1** | **1** | **1** | **0** | **0** | **1** | **10** |
| **Geetha et al.**[16] | **1** | **1** | **0** | **1** | **1** | **1** | **1** | **0** | **0** | **1** | **1** | **0** | **0** | **1** | **9** |
| **Casalegno et al.**[17] | **1** | **1** | **0** | **1** | **1** | **1** | **1** | **0** | **1** | **0** | **0** | **0** | **0** | **0** | **7** |
| **Devito et al.**[18] | **1** | **1** | **0** | **1** | **0** | **1** | **0** | **0** | **1** | **0** | **0** | **0** | **0** | **1** | **6** |
| **Cantu et al.**[19] | **0** | **1** | **0** | **0** | **1** | **0** | **1** | **0** | **0** | **0** | **1** | **1** | **0** | **1** | **6** |
| **Schwendicke et al.**[20] | **0** | **1** | **0** | **1** | **1** | **0** | **1** | **0** | **1** | **0** | **0** | **0** | **0** | **0** | **5** |
| **Moutselos et al.**[21] | **1** | **0** | **1** | **1** | **1** | **1** | **0** | **0** | **0** | **0** | **0** | **0** | **0** | **0** | **5** |
| **Moran et al.**[22] | **0** | **0** | **0** | **0** | **0** | **0** | **1** | **0** | **0** | **1** | **1** | **1** | **0** | **1** | **5** |
| **Bayrakdar et al.**[23] | **0** | **0** | **0** | **0** | **0** | **0** | **1** | **0** | **0** | **1** | **1** | **1** | **0** | **1** | **5** |
| **Lee et al.**[24] | **0** | **0** | **0** | **0** | **0** | **0** | **1** | **0** | **0** | **1** | **1** | **1** | **0** | **1** | **5** |
| **Mao et al.**[25] | **0** | **0** | **0** | **0** | **0** | **0** | **1** | **0** | **0** | **1** | **1** | **1** | **0** | **1** | **5** |
| **Zhang et al.**[26] | **0** | **1** | **1** | **0** | **1** | **0** | **1** | **0** | **0** | **0** | **0** | **0** | **0** | **0** | **4** |
| **Bayraktar et al.**[27] | **0** | **0** | **0** | **0** | **0** | **0** | **1** | **0** | **0** | **0** | **1** | **1** | **0** | **1** | **4** |
| **Lian et al.**[28] | **0** | **0** | **0** | **0** | **0** | **0** | **1** | **0** | **0** | **1** | **1** | **0** | **0** | **1** | **4** |
| **Zhu et al.**[29] | **0** | **0** | **0** | **0** | **0** | **0** | **1** | **0** | **0** | **1** | **1** | **0** | **0** | **1** | **4** |
| **Berdouses et al.**[30] | **0** | **1** | **0** | **1** | **0** | **1** | **0** | **0** | **0** | **0** | **0** | **0** | **0** | **0** | **3** |
| **Wang et al.**[31] | **0** | **1** | **1** | **0** | **1** | **0** | **0** | **0** | **0** | **0** | **0** | **0** | **0** | **0** | **3** |
| **Leo et al.**[32] | **0** | **0** | **0** | **0** | **1** | **0** | **0** | **0** | **0** | **1** | **1** | **0** | **0** | **0** | **3** |
| **Sornam et al.**[33] | **0** | **0** | **0** | **1** | **1** | **0** | **0** | **0** | **0** | **1** | **0** | **0** | **0** | **0** | **3** |
| **Srivastava et al.**[34] | **1** | **0** | **0** | **1** | **1** | **0** | **0** | **0** | **0** | **0** | **0** | **0** | **0** | **0** | **3** |
| **Choi et al.**[35] | **0** | **0** | **0** | **0** | **1** | **0** | **1** | **0** | **0** | **0** | **0** | **0** | **0** | **1** | **3** |
| **Khan et al.**[36] | **0** | **0** | **0** | **0** | **1** | **0** | **0** | **0** | **0** | **1** | **1** | **0** | **0** | **0** | **3** |
| **Chen et al.**[37] | **0** | **0** | **0** | **0** | **0** | **0** | **1** | **0** | **0** | **0** | **1** | **0** | **0** | **1** | **3** |
| **Devlin et al.**[38] | **0** | **0** | **0** | **0** | **0** | **0** | **1** | **0** | **0** | **0** | **1** | **0** | **0** | **1** | **3** |
| **Mertens et al.**[39] | **0** | **0** | **0** | **0** | **0** | **0** | **1** | **0** | **0** | **0** | **1** | **0** | **0** | **1** | **3** |
| **Panyarak et al.**[40] | **0** | **0** | **0** | **0** | **0** | **0** | **0** | **0** | **0** | **1** | **1** | **1** | **0** | **0** | **3** |
| **Chen et al.**[41] | **0** | **0** | **0** | **0** | **0** | **0** | **0** | **0** | **0** | **1** | **1** | **1** | **0** | **0** | **3** |
| **Li et al.**[42] | **0** | **0** | **0** | **0** | **0** | **0** | **0** | **0** | **0** | **1** | **1** | **0** | **0** | **1** | **3** |
| **Panyarak et al.**[43] | **0** | **0** | **0** | **0** | **0** | **0** | **0** | **0** | **0** | **1** | **1** | **1** | **0** | **0** | **3** |
| **Araki et al.**[44] | **0** | **1** | **0** | **0** | **0** | **1** | **0** | **0** | **0** | **0** | **0** | **0** | **0** | **0** | **2** |
| **Zhang et al.**[45] | **0** | **0** | **1** | **0** | **1** | **0** | **0** | **0** | **0** | **0** | **0** | **0** | **0** | **0** | **2** |
| **Javid et al.**[46] | **0** | **0** | **1** | **0** | **1** | **0** | **0** | **0** | **0** | **0** | **0** | **0** | **0** | **0** | **2** |
| **Yu et al.**[47] | **0** | **0** | **1** | **0** | **1** | **0** | **0** | **0** | **0** | **0** | **0** | **0** | **0** | **0** | **2** |
| **Sonavane et al.**[48] | **0** | **0** | **1** | **0** | **1** | **0** | **0** | **0** | **0** | **0** | **0** | **0** | **0** | **0** | **2** |
| **Askar et al.**[49] | **0** | **0** | **1** | **0** | **0** | **0** | **1** | **0** | **0** | **0** | **0** | **0** | **0** | **0** | **2** |
| **Kühnisch et al.**[50] | **1** | **0** | **1** | **0** | **0** | **0** | **0** | **0** | **0** | **0** | **0** | **0** | **0** | **0** | **2** |
| **Prajapati et al.**[51] | **0** | **0** | **0** | **1** | **1** | **0** | **0** | **0** | **0** | **0** | **0** | **0** | **0** | **0** | **2** |
| **Lakshmi et al.**[52] | **0** | **0** | **0** | **0** | **1** | **0** | **0** | **0** | **0** | **1** | **0** | **0** | **0** | **0** | **2** |
| **Singh et al.**[53] | **0** | **0** | **0** | **1** | **0** | **0** | **0** | **0** | **0** | **1** | **0** | **0** | **0** | **0** | **2** |
| **Patil et al.**[54] | **1** | **0** | **0** | **0** | **0** | **0** | **0** | **0** | **0** | **1** | **0** | **0** | **0** | **0** | **2** |
| **Duong et al.**[55] | **1** | **0** | **0** | **0** | **0** | **0** | **1** | **0** | **0** | **0** | **0** | **0** | **0** | **0** | **2** |
| **Ezhov et al.**[56] | **0** | **0** | **0** | **0** | **0** | **0** | **0** | **0** | **0** | **1** | **1** | **0** | **0** | **0** | **2** |
| **Vinayahali-ngam et al.**[57] | **0** | **0** | **0** | **0** | **0** | **0** | **1** | **0** | **0** | **0** | **0** | **0** | **0** | **1** | **2** |
| **De Araujo Faria et al.**[58] | **0** | **0** | **0** | **0** | **0** | **0** | **1** | **0** | **0** | **0** | **0** | **0** | **0** | **1** | **2** |
| **Huang et al.**[59] | **0** | **0** | **0** | **0** | **1** | **0** | **1** | **0** | **0** | **0** | **0** | **0** | **0** | **0** | **2** |
| **Baydar et al.**[60] | **0** | **0** | **0** | **0** | **0** | **0** | **0** | **0** | **0** | **1** | **0** | **1** | **0** | **0** | **2** |
| **Imak et al.**[61] | **0** | **0** | **0** | **0** | **0** | **0** | **0** | **0** | **0** | **1** | **1** | **0** | **0** | **0** | **2** |
| **Ying et al.**[62] | **0** | **0** | **0** | **0** | **0** | **0** | **0** | **0** | **0** | **1** | **1** | **0** | **0** | **0** | **2** |
| **Estai et al.**[63] | **0** | **0** | **0** | **0** | **0** | **0** | **0** | **0** | **0** | **0** | **1** | **1** | **0** | **0** | **2** |
| **Taleb et al.**[64] | **0** | **0** | **0** | **0** | **0** | **0** | **0** | **0** | **0** | **1** | **1** | **0** | **0** | **0** | **2** |
| **Kim et al.**[65] | **0** | **0** | **0** | **0** | **0** | **0** | **0** | **0** | **0** | **1** | **1** | **0** | **0** | **0** | **2** |
| **Zhu et al.**[66] | **0** | **0** | **0** | **0** | **0** | **0** | **0** | **0** | **0** | **1** | **1** | **0** | **0** | **0** | **2** |
| **Vimalarani et al.**[67] | **0** | **0** | **0** | **0** | **0** | **0** | **0** | **0** | **0** | **1** | **1** | **0** | **0** | **0** | **2** |
| **Li et al.**[68] | **0** | **0** | **0** | **0** | **0** | **0** | **0** | **1** | **0** | **0** | **0** | **0** | **1** | **0** | **2** |
| **Navarro et al.**[69] | **0** | **0** | **1** | **0** | **0** | **0** | **0** | **0** | **0** | **0** | **0** | **0** | **0** | **0** | **1** |
| **Liu et al.**[70] | **0** | **0** | **1** | **0** | **0** | **0** | **0** | **0** | **0** | **0** | **0** | **0** | **0** | **0** | **1** |
| **Kim et al.**[71] | **0** | **0** | **1** | **0** | **0** | **0** | **0** | **0** | **0** | **0** | **0** | **0** | **0** | **0** | **1** |
| **Ding et al.**[72] | **0** | **0** | **1** | **0** | **0** | **0** | **0** | **0** | **0** | **0** | **0** | **0** | **0** | **0** | **1** |
| **Jiang et al.**[73] | **0** | **0** | **1** | **0** | **0** | **0** | **0** | **0** | **0** | **0** | **0** | **0** | **0** | **0** | **1** |
| **Saini et al.**[74] | **0** | **0** | **1** | **0** | **0** | **0** | **0** | **0** | **0** | **0** | **0** | **0** | **0** | **0** | **1** |
| **Rashid et al.**[75] | **0** | **0** | **1** | **0** | **0** | **0** | **0** | **0** | **0** | **0** | **0** | **0** | **0** | **0** | **1** |
| **Park et al.**[76] | **0** | **0** | **1** | **0** | **0** | **0** | **0** | **0** | **0** | **0** | **0** | **0** | **0** | **0** | **1** |
| **Thanh et al.**[77] | **0** | **0** | **1** | **0** | **0** | **0** | **0** | **0** | **0** | **0** | **0** | **0** | **0** | **0** | **1** |
| **Khan et al.**[78] | **0** | **0** | **1** | **0** | **0** | **0** | **0** | **0** | **0** | **0** | **0** | **0** | **0** | **0** | **1** |
| **Leo et al.**[79] | **0** | **0** | **0** | **0** | **1** | **0** | **0** | **0** | **0** | **0** | **0** | **0** | **0** | **0** | **1** |
| **Tripathi et al.**[80] | **0** | **0** | **0** | **0** | **1** | **0** | **0** | **0** | **0** | **0** | **0** | **0** | **0** | **0** | **1** |
| **Singh et al.**[81] | **0** | **0** | **0** | **0** | **1** | **0** | **0** | **0** | **0** | **0** | **0** | **0** | **0** | **0** | **1** |
| **Singh et al.**[82] | **0** | **0** | **0** | **0** | **1** | **0** | **0** | **0** | **0** | **0** | **0** | **0** | **0** | **0** | **1** |
| **G-Rodríguez et al.**[83] | **0** | **0** | **0** | **0** | **1** | **0** | **0** | **0** | **0** | **0** | **0** | **0** | **0** | **0** | **1** |
| **Holtkamp et al.**[84] | **0** | **0** | **0** | **0** | **1** | **0** | **0** | **0** | **0** | **0** | **0** | **0** | **0** | **0** | **1** |
| **Salehi et al.**[85] | **0** | **0** | **0** | **0** | **1** | **0** | **0** | **0** | **0** | **0** | **0** | **0** | **0** | **0** | **1** |
| **Salehi et al.**[86] | **0** | **0** | **0** | **0** | **1** | **0** | **0** | **0** | **0** | **0** | **0** | **0** | **0** | **0** | **1** |
| **Salehi et al.**[87] | **0** | **0** | **0** | **0** | **1** | **0** | **0** | **0** | **0** | **0** | **0** | **0** | **0** | **0** | **1** |
| **Riyadi et al.**[88] | **0** | **0** | **0** | **0** | **1** | **0** | **0** | **0** | **0** | **0** | **0** | **0** | **0** | **0** | **1** |
| **Patil et al.**[89] | **0** | **0** | **0** | **0** | **1** | **0** | **0** | **0** | **0** | **0** | **0** | **0** | **0** | **0** | **1** |
| **Sornam et al.**[90] | **0** | **0** | **0** | **0** | **1** | **0** | **0** | **0** | **0** | **0** | **0** | **0** | **0** | **0** | **1** |
| **Patil et al.**[91] | **0** | **0** | **0** | **0** | **1** | **0** | **0** | **0** | **0** | **0** | **0** | **0** | **0** | **0** | **1** |
| **Patil et al.**[92] | **0** | **0** | **0** | **0** | **1** | **0** | **0** | **0** | **0** | **0** | **0** | **0** | **0** | **0** | **1** |
| **Vinayahali-ngam et al.**[93] | **0** | **0** | **0** | **0** | **1** | **0** | **0** | **0** | **0** | **0** | **0** | **0** | **0** | **0** | **1** |
| **Haghanifar et al.**[94] | **0** | **0** | **0** | **0** | **1** | **0** | **0** | **0** | **0** | **0** | **0** | **0** | **0** | **0** | **1** |
| **Kumar et al.**[95] | **0** | **0** | **0** | **0** | **1** | **0** | **0** | **0** | **0** | **0** | **0** | **0** | **0** | **0** | **1** |
| **Yun et al.**[96] | **0** | **0** | **0** | **0** | **1** | **0** | **0** | **0** | **0** | **0** | **0** | **0** | **0** | **0** | **1** |
| **Ronneberg-er et al.**[97] | **0** | **0** | **0** | **0** | **1** | **0** | **0** | **0** | **0** | **0** | **0** | **0** | **0** | **0** | **1** |
| **Ezhov et al.**[98] | **0** | **0** | **0** | **0** | **1** | **0** | **0** | **0** | **0** | **0** | **0** | **0** | **0** | **0** | **1** |
| **Jung et al.**[99] | **0** | **0** | **0** | **0** | **1** | **0** | **0** | **0** | **0** | **0** | **0** | **0** | **0** | **0** | **1** |
| **Rad et al.**[100] | **0** | **0** | **0** | **0** | **1** | **0** | **0** | **0** | **0** | **0** | **0** | **0** | **0** | **0** | **1** |
| **Kuang et al.**[101] | **0** | **0** | **0** | **1** | **0** | **0** | **0** | **0** | **0** | **0** | **0** | **0** | **0** | **0** | **1** |
| **Javed et al.**[102] | **1** | **0** | **0** | **0** | **0** | **0** | **0** | **0** | **0** | **0** | **0** | **0** | **0** | **0** | **1** |
| **Valizadeh et al.**[103] | **0** | **0** | **0** | **0** | **0** | **1** | **0** | **0** | **0** | **0** | **0** | **0** | **0** | **0** | **1** |
| **Duong et al.**[104] | **0** | **0** | **0** | **0** | **0** | **0** | **1** | **0** | **0** | **0** | **0** | **0** | **0** | **0** | **1** |
| **Zheng et al.**[105] | **0** | **0** | **0** | **0** | **0** | **0** | **1** | **0** | **0** | **0** | **0** | **0** | **0** | **0** | **1** |
| **Oztekin et al.**[106] | **0** | **0** | **0** | **0** | **0** | **0** | **0** | **0** | **0** | **1** | **0** | **0** | **0** | **0** | **1** |
| **Canas et al.**[107] | **0** | **0** | **0** | **0** | **0** | **0** | **0** | **0** | **0** | **0** | **1** | **0** | **0** | **0** | **1** |
| **Pitts et al.**[108] | **0** | **0** | **0** | **0** | **0** | **1** | **0** | **0** | **0** | **0** | **0** | **0** | **0** | **0** | **1** |
| **Pitts et al.**[109] | **0** | **0** | **0** | **0** | **0** | **1** | **0** | **0** | **0** | **0** | **0** | **0** | **0** | **0** | **1** |
| **Pitts et al.**[110] | **0** | **0** | **0** | **0** | **0** | **1** | **0** | **0** | **0** | **0** | **0** | **0** | **0** | **0** | **1** |
| **Pitts et al.**[111] | **0** | **0** | **0** | **0** | **0** | **1** | **0** | **0** | **0** | **0** | **0** | **0** | **0** | **0** | **1** |
| **Pitts et al.**[112] | **0** | **0** | **0** | **0** | **0** | **1** | **0** | **0** | **0** | **0** | **0** | **0** | **0** | **0** | **1** |
| **Heaven et al.**[113] | **0** | **0** | **0** | **0** | **0** | **1** | **0** | **0** | **0** | **0** | **0** | **0** | **0** | **0** | **1** |
| **Heaven et al.**[114] | **0** | **0** | **0** | **0** | **0** | **1** | **0** | **0** | **0** | **0** | **0** | **0** | **0** | **0** | **1** |
| **Duncan et al.**[115] | **0** | **0** | **0** | **0** | **0** | **1** | **0** | **0** | **0** | **0** | **0** | **0** | **0** | **0** | **1** |
| **Firestone et al.**[116] | **0** | **0** | **0** | **0** | **0** | **1** | **0** | **0** | **0** | **0** | **0** | **0** | **0** | **0** | **1** |
| **Son et al.**[117] | **0** | **0** | **0** | **0** | **0** | **1** | **0** | **0** | **0** | **0** | **0** | **0** | **0** | **0** | **1** |
| **Ghaedi et al.**[118] | **0** | **0** | **0** | **0** | **0** | **1** | **0** | **0** | **0** | **0** | **0** | **0** | **0** | **0** | **1** |
| **Berdouses et al.**[119] | **0** | **0** | **0** | **0** | **0** | **1** | **0** | **0** | **0** | **0** | **0** | **0** | **0** | **0** | **1** |
| **Rahman et al.**[120] | **0** | **0** | **0** | **0** | **0** | **1** | **0** | **0** | **0** | **0** | **0** | **0** | **0** | **0** | **1** |
| **Gakenheim-er et al.**[121] | **0** | **0** | **0** | **0** | **0** | **1** | **0** | **0** | **0** | **0** | **0** | **0** | **0** | **0** | **1** |
| **Wenzel et al.**[122] | **0** | **0** | **0** | **0** | **0** | **1** | **0** | **0** | **0** | **0** | **0** | **0** | **0** | **0** | **1** |
| **Forner et al.**[123] | **0** | **0** | **0** | **0** | **0** | **1** | **0** | **0** | **0** | **0** | **0** | **0** | **0** | **0** | **1** |
| **Moutselos et al.**[124] | **0** | **0** | **0** | **0** | **0** | **1** | **0** | **0** | **0** | **0** | **0** | **0** | **0** | **0** | **1** |
| **Udod et al.**[125] | **0** | **0** | **0** | **0** | **0** | **1** | **0** | **0** | **0** | **0** | **0** | **0** | **0** | **0** | **1** |
| **Bhan et al.**[126] | **0** | **0** | **0** | **0** | **0** | **0** | **0** | **0** | **0** | **1** | **0** | **0** | **0** | **0** | **1** |
| **Naebi et al.**[127] | **0** | **0** | **0** | **0** | **0** | **0** | **0** | **0** | **0** | **1** | **0** | **0** | **0** | **0** | **1** |
| **Datta et al.**[128] | **0** | **0** | **0** | **0** | **0** | **0** | **0** | **0** | **0** | **1** | **0** | **0** | **0** | **0** | **1** |
| **Al Kheraif et al.**[129] | **0** | **0** | **0** | **0** | **0** | **0** | **0** | **0** | **0** | **1** | **0** | **0** | **0** | **0** | **1** |
| **Verma et al.**[130] | **0** | **0** | **0** | **0** | **0** | **0** | **0** | **0** | **0** | **1** | **0** | **0** | **0** | **0** | **1** |
| **Jusman et al.**[131] | **0** | **0** | **0** | **0** | **0** | **0** | **0** | **0** | **0** | **1** | **0** | **0** | **0** | **0** | **1** |
| **Choudhary et al.**[132] | **0** | **0** | **0** | **0** | **0** | **0** | **0** | **0** | **0** | **1** | **0** | **0** | **0** | **0** | **1** |
| **Fariza et al.**[133] | **0** | **0** | **0** | **0** | **0** | **0** | **0** | **0** | **0** | **1** | **0** | **0** | **0** | **0** | **1** |
| **Jusman et al.**[134] | **0** | **0** | **0** | **0** | **0** | **0** | **0** | **0** | **0** | **1** | **0** | **0** | **0** | **0** | **1** |
| **Jusman et al.**[135] | **0** | **0** | **0** | **0** | **0** | **0** | **0** | **0** | **0** | **1** | **0** | **0** | **0** | **0** | **1** |
| **Jayasinghe et al.**[136] | **0** | **0** | **0** | **0** | **0** | **0** | **0** | **0** | **0** | **1** | **0** | **0** | **0** | **0** | **1** |
| **Liu et al.**[137] | **0** | **0** | **0** | **0** | **0** | **0** | **0** | **0** | **0** | **1** | **0** | **0** | **0** | **0** | **1** |
| **Kumari et al.**[138] | **0** | **0** | **0** | **0** | **0** | **0** | **0** | **0** | **0** | **1** | **0** | **0** | **0** | **0** | **1** |
| **Dayi et al.**[139] | **0** | **0** | **0** | **0** | **0** | **0** | **0** | **0** | **0** | **1** | **0** | **0** | **0** | **0** | **1** |
| **Lin et al.**[140] | **0** | **0** | **0** | **0** | **0** | **0** | **0** | **0** | **0** | **0** | **1** | **0** | **0** | **0** | **1** |
| **Majanga et al.**[141] | **0** | **0** | **0** | **0** | **0** | **0** | **0** | **0** | **0** | **0** | **1** | **0** | **0** | **0** | **1** |
| **Bui et al.**[142] | **0** | **0** | **0** | **0** | **0** | **0** | **0** | **0** | **0** | **0** | **1** | **0** | **0** | **0** | **1** |
| **Ari et al.**[143] | **0** | **0** | **0** | **0** | **0** | **0** | **0** | **0** | **0** | **0** | **1** | **0** | **0** | **0** | **1** |
| **Almalki et al.**[144] | **0** | **0** | **0** | **0** | **0** | **0** | **0** | **0** | **0** | **0** | **1** | **0** | **0** | **0** | **1** |
| **Bui et al.**[145] | **0** | **0** | **0** | **0** | **0** | **0** | **0** | **0** | **0** | **0** | **1** | **0** | **0** | **0** | **1** |
| **Chen et al.**[146] | **0** | **0** | **0** | **0** | **0** | **0** | **0** | **0** | **0** | **0** | **1** | **0** | **0** | **0** | **1** |
| **Panyarak et al.**[147] | **0** | **0** | **0** | **0** | **0** | **0** | **0** | **0** | **0** | **0** | **0** | **1** | **0** | **0** | **1** |
| **Suttapak et al.**[148] | **0** | **0** | **0** | **0** | **0** | **0** | **0** | **0** | **0** | **0** | **0** | **1** | **0** | **0** | **1** |
| **Ahmed et al.**[149] | **0** | **0** | **0** | **0** | **0** | **0** | **0** | **0** | **0** | **0** | **0** | **1** | **0** | **0** | **1** |
| **Zadrożny et al.**[150] | **0** | **0** | **0** | **0** | **0** | **0** | **0** | **0** | **0** | **0** | **0** | **0** | **0** | **1** | **1** |
| **Al-Jallad et al.**[151] | **0** | **0** | **0** | **0** | **0** | **0** | **0** | **0** | **0** | **0** | **0** | **0** | **1** | **0** | **1** |

**References**

1. Talpur S, Azim F, Rashid M, Syed SA, Talpur BA, Khan SJ. Uses of Different Machine Learning Algorithms for Diagnosis of Dental Caries. J Healthc Eng. 2022;2022. doi:10.1155/2022/5032435

2. Reyes LT, Knorst JK, Ortiz FR, Ardenghi TM. Machine Learning in the Diagnosis and Prognostic Prediction of Dental Caries: A Systematic Review. Caries Res. 20220530th ed. 2022;56: 161–170. doi:10.1159/000524167

3. Moharrami M, Farmer J, Singhal S, Watson E, Glogauer M, Johnson AEW, et al. Detecting dental caries on oral photographs using artificial intelligence: A systematic review. Oral Dis. 20230701st ed. 2023. doi:10.1111/odi.14659

4. Prados-Privado M, García Villalón J, Martínez-Martínez CH, Ivorra C, Prados-Frutos JC. Dental Caries Diagnosis and Detection Using Neural Networks: A Systematic Review. J Clin Med. 20201106th ed. 2020;9. doi:10.3390/jcm9113579

5. Mohammad-Rahimi H, Motamedian SR, Rohban MH, Krois J, Uribe SE, Mahmoudinia E, et al. Deep learning for caries detection: A systematic review. J Dent. 20220330th ed. 2022;122: 104115. doi:10.1016/j.jdent.2022.104115

6. Revilla-León M, Gómez-Polo M, Vyas S, Barmak AB, Özcan M, Att W, et al. Artificial intelligence applications in restorative dentistry: A systematic review. J Prosthet Dent. 2022;128: 867–875. doi:10.1016/j.prosdent.2021.02.010

7. Khanagar SB, Alfouzan K, Awawdeh M, Alkadi L, Albalawi F, Alfadley A. Application and Performance of Artificial Intelligence Technology in Detection, Diagnosis and Prediction of Dental Caries (DC)—A Systematic Review. Diagnostics. 2022;12: 1083. doi:10.3390/diagnostics12051083

8. Khanagar SB, Alfouzan K, Alkadi L, Albalawi F, Iyer K, Awawdeh M. Performance of Artificial Intelligence (AI) Models Designed for Application in Pediatric Dentistry—A Systematic Review. Applied Sciences. 2022;12: 9819. doi:10.3390/app12199819

9. Khanagar SB, Alfadley A, Alfouzan K, Awawdeh M, Alaqla A, Jamleh A. Developments and Performance of Artificial Intelligence Models Designed for Application in Endodontics: A Systematic Review. Diagnostics (Basel). 20230123rd ed. 2023;13. doi:10.3390/diagnostics13030414

10. Zanini LGK, Rubira-Bullen IRF, Nunes F de L dos S. A Systematic Review on Caries Detection, Classification, and Segmentation from X-Ray Images: Methods, Datasets, Evaluation, and Open Opportunities. Journal of Imaging Informatics in Medicine. 2024. doi:10.1007/s10278-024-01054-5

11. Ndiaye AD, Gasqui MA, Millioz F, Perard M, Leye Benoist F, Grosgogeat B. Exploring the Methodological Approaches of Studies on Radiographic Databases Used in Cariology to Feed Artificial Intelligence: A Systematic Review. Caries Res. 2024; 1–24. doi:10.1159/000536277

12. Ammar N, Kühnisch J. Diagnostic performance of artificial intelligence-aided caries detection on bitewing radiographs: a systematic review and meta-analysis. Japanese Dental Science Review. 2024;60: 128–136. doi:10.1016/j.jdsr.2024.02.001

13. Rokhshad R, Zhang P, Mohammad-Rahimi H, Shobeiri P, Schwendicke F. Current Applications of Artificial Intelligence for Pediatric Dentistry: A Systematic Review and Meta-Analysis. Pediatr Dent. 2024;46: 27–35.

14. Albano D, Galiano V, Basile M, Di Luca F, Gitto S, Messina C, et al. Artificial intelligence for radiographic imaging detection of caries lesions: a systematic review. BMC Oral Health. 2024;24: 274. doi:10.1186/s12903-024-04046-7

15. Lee J-H, Kim D-H, Jeong S-N, Choi S-H. Detection and diagnosis of dental caries using a deep learning-based convolutional neural network algorithm. J Dent. 2018;77: 106–111. doi:10.1016/j.jdent.2018.07.015

16. Geetha V, Aprameya KS, Hinduja DM. Dental caries diagnosis in digital radiographs using back-propagation neural network. Health Inf Sci Syst. 2020;8: 8. doi:10.1007/s13755-019-0096-y

17. Casalegno F, Newton T, Daher R, Abdelaziz M, Lodi-Rizzini A, Schürmann F, et al. Caries Detection with Near-Infrared Transillumination Using Deep Learning. J Dent Res. 2019;98: 1227–1233. doi:10.1177/0022034519871884

18. Devito KL, de Souza Barbosa F, Filho WNF. An artificial multilayer perceptron neural network for diagnosis of proximal dental caries. Oral Surgery, Oral Medicine, Oral Pathology, Oral Radiology, and Endodontology. 2008;106: 879–884. doi:10.1016/j.tripleo.2008.03.002

19. Cantu AG, Gehrung S, Krois J, Chaurasia A, Rossi JG, Gaudin R, et al. Detecting caries lesions of different radiographic extension on bitewings using deep learning. J Dent. 2020;100: 103425. doi:10.1016/j.jdent.2020.103425

20. Schwendicke F, Elhennawy K, Paris S, Friebertshäuser P, Krois J. Deep learning for caries lesion detection in near-infrared light transillumination images: A pilot study. J Dent. 2020;92: 103260. doi:10.1016/j.jdent.2019.103260

21. Moutselos K, Berdouses E, Oulis C, Maglogiannis I. Recognizing Occlusal Caries in Dental Intraoral Images Using Deep Learning. 2019 41st Annual International Conference of the IEEE Engineering in Medicine and Biology Society (EMBC). IEEE; 2019. pp. 1617–1620. doi:10.1109/EMBC.2019.8856553

22. Moran M, Faria M, Giraldi G, Bastos L, Oliveira L, Conci A. Classification of Approximal Caries in Bitewing Radiographs Using Convolutional Neural Networks. Sensors. 2021;21: 5192. doi:10.3390/s21155192

23. Lee S, Oh S, Jo J, Kang S, Shin Y, Park J. Deep learning for early dental caries detection in bitewing radiographs. Sci Rep. 2021;11: 16807. doi:10.1038/s41598-021-96368-7

24. Mao Y-C, Chen T-Y, Chou H-S, Lin S-Y, Liu S-Y, Chen Y-A, et al. Caries and Restoration Detection Using Bitewing Film Based on Transfer Learning with CNNs. Sensors. 2021;21. doi:10.3390/s21134613

25. Zhang X, Liang Y, Li W, Liu C, Gu D, Sun W, et al. Development and evaluation of deep learning for screening dental caries from oral photographs. Oral Dis. 2022;28: 173–181. doi:10.1111/odi.13735

26. Bayraktar Y, Ayan E. Diagnosis of interproximal caries lesions with deep convolutional neural network in digital bitewing radiographs. Clin Oral Investig. 2022;26: 623–632. doi:10.1007/s00784-021-04040-1

27. Lian L, Zhu T, Zhu F, Zhu H. Deep Learning for Caries Detection and Classification. Diagnostics. 2021;11. doi:10.3390/diagnostics11091672

28. Zhu H, Cao Z, Lian L, Ye G, Gao H, Wu J. CariesNet: a deep learning approach for segmentation of multi-stage caries lesion from oral panoramic X-ray image. Neural Comput Appl. 2023;35: 16051–16059. doi:10.1007/s00521-021-06684-2

29. Berdouses ED, Koutsouri GD, Tripoliti EE, Matsopoulos GK, Oulis CJ, Fotiadis DI. A computer-aided automated methodology for the detection and classification of occlusal caries from photographic color images. Comput Biol Med. 2015;62: 119–135. doi:10.1016/j.compbiomed.2015.04.016

30. Wang C, Qin H, Lai G, Zheng G, Xiang H, Wang J, et al. Automated classification of dual channel dental imaging of auto-fluorescence and white lightby convolutional neural networks. J Innov Opt Health Sci. 2020;13. doi:10.1142/S1793545820500145

31. Megalan Leo L, Kalapalatha Reddy T. Learning compact and discriminative hybrid neural network for dental caries classification. Microprocess Microsyst. 2021;82: 103836. doi:https://doi.org/10.1016/j.micpro.2021.103836

32. Sornam M, Prabhakaran M. A new linear adaptive swarm intelligence approach using back propagation neural network for dental caries classification. 2017 IEEE International Conference on Power, Control, Signals and Instrumentation Engineering (ICPCSI). 2017. pp. 2698–2703. doi:10.1109/ICPCSI.2017.8392208

33. Srivastava MM, Kumar P, Pradhan L, Varadarajan S. Detection of Tooth caries in Bitewing Radiographs using Deep Learning. 2017.

34. Choi J, Eun H, Kim C. Boosting Proximal Dental Caries Detection via Combination of Variational Methods and Convolutional Neural Network. J Signal Process Syst. 2018;90: 87–97. doi:10.1007/s11265-016-1214-6

35. Khan HA, Haider MA, Ansari HA, Ishaq H, Kiyani A, Sohail K, et al. Automated feature detection in dental periapical radiographs by using deep learning. Oral Surg Oral Med Oral Pathol Oral Radiol. 2021;131: 711–720. doi:10.1016/j.oooo.2020.08.024

36. Chen H, Li H, Zhao Y, Zhao J, Wang Y. Dental disease detection on periapical radiographs based on deep convolutional neural networks. Int J Comput Assist Radiol Surg. 2021;16: 649–661. doi:10.1007/s11548-021-02319-y

37. Devlin H, Williams T, Graham J, Ashley M. The ADEPT study: a comparative study of dentists’ ability to detect enamel-only proximal caries in bitewing radiographs with and without the use of AssistDent artificial intelligence software. Br Dent J. 2021;231: 481–485. doi:10.1038/s41415-021-3526-6

38. Mertens S, Krois J, Cantu AG, Arsiwala LT, Schwendicke F. Artificial intelligence for caries detection: Randomized trial. J Dent. 2021;115: 103849. doi:https://doi.org/10.1016/j.jdent.2021.103849

39. Panyarak W, Suttapak W, Wantanajittikul K, Charuakkra A, Prapayasatok S. Correction to: Assessment of YOLOv3 for caries detection in bitewing radiographs based on the ICCMS^TM^ radiographic scoring system. Clin Oral Investig. 2023;27: 1743. doi:10.1007/s00784-023-04865-y

40. Chen X, Guo J, Ye J, Zhang M, Liang Y. Detection of Proximal Caries Lesions on Bitewing Radiographs Using Deep Learning Method. Caries Res. 2022;56: 455–463. doi:10.1159/000527418

41. Li S, Liu J, Zhou Z, Zhou Z, Wu X, Li Y, et al. Artificial intelligence for caries and periapical periodontitis detection. J Dent. 2022;122: 104107. doi:10.1016/j.jdent.2022.104107

42. Panyarak W, Wantanajittikul K, Suttapak W, Charuakkra A, Prapayasatok S. Feasibility of deep learning for dental caries classification in bitewing radiographs based on the ICCMS^TM^ radiographic scoring system. Oral Surg Oral Med Oral Pathol Oral Radiol. 2023;135: 272–281. doi:10.1016/j.oooo.2022.06.012

43. Araki K, Matsuda Y, Seki K, Okano T. Effect of computer assistance on observer performance of approximal caries diagnosis using intraoral digital radiography. Clin Oral Investig. 2010;14: 319–325. doi:10.1007/s00784-009-0307-z

44. Zhang Y, Liao H, Xiao J, Jallad N Al, Ly-Mapes O, Luo J. A Smartphone-based System for Real-time Early Childhood Caries Diagnosis. ASMUS/PIPPI@MICCAI. 2020. Available: https://api.semanticscholar.org/CorpusID:221151024

45. Javid A, Rashid U, Khattak AS. Marking Early Lesions in Labial Colored Dental Images using a Transfer Learning Approach. 2020 IEEE 23rd International Multitopic Conference (INMIC). 2020; 1–5. Available: https://api.semanticscholar.org/CorpusID:231684587

46. Yu H, Lin Z, Liu Y, Su J, Chen B, Lu G. A New Technique for Diagnosis of Dental Caries on the Children’s First Permanent Molar. IEEE Access. 2020;8: 185776–185785. doi:10.1109/ACCESS.2020.3029454

47. Sonavane A, Yadav R, Khamparia A. Dental cavity Classification of using Convolutional Neural Network. IOP Conf Ser Mater Sci Eng. 2021;1022: 012116. doi:10.1088/1757-899X/1022/1/012116

48. Askar H, Krois J, Rohrer C, Mertens S, Elhennawy K, Ottolenghi L, et al. Detecting white spot lesions on dental photography using deep learning: A pilot study. J Dent. 2021;107: 103615. doi:10.1016/j.jdent.2021.103615

49. Kühnisch J, Meyer O, Hesenius M, Hickel R, Gruhn V. Caries Detection on Intraoral Images Using Artificial Intelligence. J Dent Res. 2022;101: 158–165. doi:10.1177/00220345211032524

50. Prajapati SA, Nagaraj R, Mitra S. Classification of dental diseases using CNN and transfer learning. 2017 5th International Symposium on Computational and Business Intelligence (ISCBI). 2017. pp. 70–74. doi:10.1109/ISCBI.2017.8053547

51. Lakshmi MM, Chitra P. Classification of Dental Cavities from X-ray images using Deep CNN algorithm. 2020 4th International Conference on Trends in Electronics and Informatics (ICOEI)(48184). 2020. pp. 774–779. doi:10.1109/ICOEI48184.2020.9143013

52. Singh P, Sehgal P. Automated caries detection based on Radon transformation and DCT. 2017 8th International Conference on Computing, Communication and Networking Technologies (ICCCNT). 2017; 1–6. Available: https://api.semanticscholar.org/CorpusID:41389715

53. Patil S, Kulkarni V, Bhise A. Algorithmic analysis for dental caries detection using an adaptive neural network architecture. Heliyon. 2019;5: e01579. doi:10.1016/j.heliyon.2019.e01579

54. Duong DL, Kabir MH, Kuo RF. Automated caries detection with smartphone color photography using machine learning. Health Informatics J. 2021;27: 14604582211007530. doi:10.1177/14604582211007530

55. Ezhov M, Gusarev M, Golitsyna M, Yates JM, Kushnerev E, Tamimi D, et al. Clinically applicable artificial intelligence system for dental diagnosis with CBCT. Sci Rep. 2021;11: 15006. doi:10.1038/s41598-021-94093-9

56. Vinayahalingam S, Kempers S, Limon L, Deibel D, Maal T, Hanisch M, et al. Classification of caries in third molars on panoramic radiographs using deep learning. Sci Rep. 2021;11: 12609. doi:10.1038/s41598-021-92121-2

57. De Araujo Faria V, Azimbagirad M, Viani Arruda G, Fernandes Pavoni J, Cezar Felipe J, dos Santos EMCMF, et al. Prediction of Radiation-Related Dental Caries Through PyRadiomics Features and Artificial Neural Network on Panoramic Radiography. J Digit Imaging. 2021;34: 1237–1248. doi:10.1007/s10278-021-00487-6

58. Huang Y-P, Lee S-Y. Deep Learning for Caries Detection using Optical Coherence Tomography. medRxiv. 2021. doi:10.1101/2021.05.04.21256502

59. Baydar O, Różyło-Kalinowska I, Futyma-Gąbka K, Sağlam H. The U-Net Approaches to Evaluation of Dental Bite-Wing Radiographs: An Artificial Intelligence Study. Diagnostics. 2023;13. doi:10.3390/diagnostics13030453

60. Imak A, Celebi A, Siddique K, Turkoglu M, Sengur A, Salam I. Dental Caries Detection Using Score-Based Multi-Input Deep Convolutional Neural Network. IEEE Access. 2022;10: 18320–18329. doi:10.1109/ACCESS.2022.3150358

61. Ying S, Wang B, Zhu H, Liu W, Huang F. Caries segmentation on tooth X-ray images with a deep network. J Dent. 2022;119: 104076. doi:10.1016/j.jdent.2022.104076

62. Estai M, Tennant M, Gebauer D, Brostek A, Vignarajan J, Mehdizadeh M, et al. Evaluation of a deep learning system for automatic detection of proximal surface dental caries on bitewing radiographs. Oral Surg Oral Med Oral Pathol Oral Radiol. 2022;134: 262–270. doi:10.1016/j.oooo.2022.03.008

63. Taleb A, Rohrer C, Bergner B, De Leon G, Rodrigues JA, Schwendicke F, et al. Self-Supervised Learning Methods for Label-Efficient Dental Caries Classification. Diagnostics. 2022;12: 1237. doi:10.3390/diagnostics12051237

64. Kim C, Jeong H, Park W, Kim D. Tooth-Related Disease Detection System Based on Panoramic Images and Optimization Through Automation: Development Study. JMIR Med Inform. 2022;10: e38640. doi:10.2196/38640

65. Zhu Y, Xu T, Peng L, Cao Y, Zhao X, Li S, et al. Faster-RCNN based intelligent detection and localization of dental caries. Displays. 2022;74: 102201. doi:10.1016/j.displa.2022.102201

66. Vimalarani G, Ramachandraiah U. Automatic diagnosis and detection of dental caries in bitewing radiographs using pervasive deep gradient based LeNet classifier model. Microprocess Microsyst. 2022;94: 104654. doi:10.1016/j.micpro.2022.104654

67. Li RZ, Zhu JX, Wang YY, Zhao SY, Peng CF, Zhou Q, et al. [Development of a deep learning based prototype artificial intelligence system for the detection of dental caries in children]. Zhonghua Kou Qiang Yi Xue Za Zhi. 2021;56: 1253–1260. doi:10.3760/cma.j.cn112144-20210712-00323

68. Navarro PK, Cadongonan JK, Reyes M, Goma JC De. Detecting Smooth Surface Dental Caries in Frontal Teeth Using Image Processing. Proceedings of the 2019 3rd High Performance Computing and Cluster Technologies Conference. 2019. Available: https://api.semanticscholar.org/CorpusID:201102887

69. Liu L, Xu J, Huan Y, Zou Z, Yeh S-C, Zheng L-R. A Smart Dental Health-IoT Platform Based on Intelligent Hardware, Deep Learning, and Mobile Terminal. IEEE J Biomed Health Inform. 2020;24: 898–906. doi:10.1109/JBHI.2019.2919916

70. Kim D, Choi J, Ahn S, Park E. A smart home dental care system: integration of deep learning, image sensors, and mobile controller. J Ambient Intell Humaniz Comput. 2023;14: 1123–1131. doi:10.1007/s12652-021-03366-8

71. Ding B, Zhang Z, Liang Y, Wang W, Hao S, Meng Z, et al. Detection of dental caries in oral photographs taken by mobile phones based on the YOLOv3 algorithm. Ann Transl Med. 2021;9: 1622–1622. doi:10.21037/atm-21-4805

72. Jiang H, Zhang P, Che C, Jin B. RDFNet: A Fast Caries Detection Method Incorporating Transformer Mechanism. Comput Math Methods Med. 2021;2021: 1–9. doi:10.1155/2021/9773917

73. Saini D, Jain R, Thakur A. Dental Caries early detection using Convolutional Neural Network for Tele dentistry. 2021 7th International Conference on Advanced Computing and Communication Systems (ICACCS). 2021;1: 958–963. Available: https://api.semanticscholar.org/CorpusID:235339517

74. Rashid U, Javid A, Khan AR, Liu L, Ahmed A, Khalid O, et al. A hybrid mask RCNN-based tool to localize dental cavities from real-time mixed photographic images. PeerJ Comput Sci. 2022;8: e888. doi:10.7717/peerj-cs.888

75. Park EY, Cho H, Kang S, Jeong S, Kim E-K. Caries detection with tooth surface segmentation on intraoral photographic images using deep learning. BMC Oral Health. 2022;22: 573. doi:10.1186/s12903-022-02589-1

76. Thanh MTG, Van Toan N, Ngoc VTN, Tra NT, Giap CN, Nguyen DM. Deep Learning Application in Dental Caries Detection Using Intraoral Photos Taken by Smartphones. Applied Sciences. 2022;12. doi:10.3390/app12115504

77. Khan MAH, Giri PS, Jothi JAA. Detection of Cavities from Oral Images using Convolutional Neural Networks. 2022 International Conference on Electrical, Computer and Energy Technologies (ICECET). IEEE; 2022. pp. 1–6. doi:10.1109/ICECET55527.2022.9872786

78. Megalan Leo L, Kalpalatha Reddy T. Dental Caries Classification System Using Deep Learning Based Convolutional Neural Network. J Comput Theor Nanosci. 2020;17: 4660–4665. doi:10.1166/jctn.2020.9295

79. Tripathi P, Malathy C, Prabhakaran M. Genetic algorithms based approach for dental caries detection using back propagation neural network. International Journal of Recent Technology and Engineering. 2019;8: 317–319.

80. Singh P, Sehgal P. G.V Black dental caries classification and preparation technique using optimal CNN-LSTM classifier. Multimed Tools Appl. 2021;80: 5255–5272. doi:10.1007/s11042-020-09891-6

81. Singh P, Sehgal P. Decision Support System for Black Classification of Dental Images Using GIST Descriptors. In: Pati B, Panigrahi CR, Buyya R, Li K-C, editors. Advanced Computing and Intelligent Engineering. Singapore: Springer Singapore; 2020. pp. 343–352.

82. Guijarro-Rodríguez AA, Witt-Rodríguez PM, Cevallos-Torres LJ, Contreras-Puco SF, Ortiz-Zambrano MC, Torres-Martínez DE. Image Segmentation Techniques Application for the Diagnosis of Dental Caries. In: Botto-Tobar M, León-Acurio J, Díaz Cadena A, Montiel Díaz P, editors. Advances in Emerging Trends and Technologies. Cham: Springer International Publishing; 2020. pp. 312–322.

83. Holtkamp A, Elhennawy K, de Oro JE, Krois J, Paris S, Schwendicke F. Generalizability of Deep Learning Models for Caries Detection in Near-Infrared Light Transillumination Images. J Clin Med. 2021;10. doi:10.3390/jcm10050961

84. Salehi HS, Barchini M, Chen Q, Mahdian M. Toward development of automated grading system for carious lesions classification using deep learning and OCT imaging. ProcSPIE. 2021. p. 1160014. doi:10.1117/12.2581318

85. Salehi HS, Barchini M, Mahdian M. Optimization methods for deep neural networks classifying OCT images to detect dental caries. ProcSPIE. 2020. p. 112170G. doi:10.1117/12.2545421

86. Salehi HS, Mahdian M, Murshid MM, Judex S, Tadinada A. Deep learning-based quantitative analysis of dental caries using optical coherence tomography: an ex vivo study. ProcSPIE. 2019. p. 108570H. doi:10.1117/12.2510076

87. Riyadi S, Mayanti S, Damarjati C, Puspita S. Deep Learning for Pixel-Based Edge Models Classification of Tertiary Dentine Images. Proceedings of the 3rd International Conference on Information Science and Systems. New York, NY, USA: Association for Computing Machinery; 2020. pp. 57–60. doi:10.1145/3388176.3388197

88. Patil S, Kulkarni V, Bhise A. Intelligent system with dragonfly optimisation for caries detection. IET Image Process. 2019;13: 429–439. doi:https://doi.org/10.1049/iet-ipr.2018.5442

89. Sornam M, Prabhakaran M. Logit-Based Artificial Bee Colony Optimization (LB-ABC) Approach for Dental Caries Classification Using a Back Propagation Neural Network. In: Krishna AN, Srikantaiah KC, Naveena C, editors. Integrated Intelligent Computing, Communication and Security. Singapore: Springer Singapore; 2019. pp. 79–91. doi:10.1007/978-981-10-8797-4_9

90. Patil S, Kulkarni V, Bhise A. Caries detection using multidimensional projection and neural network. International Journal of Knowledge-based and Intelligent Engineering Systems. 2018;22: 155–166. doi:10.3233/KES-180381

91. Patil S, Kulkarni V, Bhise A. Caries Detection with the Aid of Multilinear Principal Component Analysis and Neural Network. 2018 Second International Conference on Green Computing and Internet of Things (ICGCIoT). 2018. pp. 272–277. doi:10.1109/ICGCIoT.2018.8753002

92. Vinayahalingam S, Kempers S, Limon L, Deibel D, Maal T, Bergé S, et al. The Automatic Detection of Caries in Third Molars on Panoramic Radiographs Using Deep Learning: A Pilot Study. 2021. doi:10.21203/rs.3.rs-379636/v1

93. Haghanifar A, Majdabadi MM, Ko S-B. PaXNet: Dental Caries Detection in Panoramic X-ray using Ensemble Transfer Learning and Capsule Classifier. 2020.

94. Kumar P, Srivastava MM. Example Mining for Incremental Learning in Medical Imaging. 2018 IEEE Symposium Series on Computational Intelligence (SSCI). 2018. pp. 48–51. doi:10.1109/SSCI.2018.8628895

95. Jiang Y, Tan N, Zhang H, Peng T. Bitewing Radiography Semantic Segmentation Base on Conditional Generative Adversarial Nets. ArXiv. 2018;abs/1802.02571. Available: https://api.semanticscholar.org/CorpusID:3652310

96. Ronneberger O, Fischer P, Brox T. Dental X-ray segmentation using a U-shaped deep learning convolutional network. International Symposium on Biomedical Imaging. 2015.

97. Ezhov M, Gusarev M, Golitsyna M, Yates J, Kushnerev E, Tamimi D, et al. Development and Validation of a Cbct-Based Artificial Intelligence System for Accurate Diagnoses of Dental Diseases. 2021. doi:10.21203/rs.3.rs-303329/v1

98. Jung Y-J, Kim M-J. Deeplab v3+ Based Automatic Diagnosis Model for Dental X-ray: Preliminary Study. Journal of Magnetics. 2020;25: 632–638. doi:10.4283/JMAG.2020.25.4.632

99. Rad AE, Rahim MSM, Kolivand H, Norouzi A. Automatic computer-aided caries detection from dental x-ray images using intelligent level set. Multimed Tools Appl. 2018;77: 28843–28862. doi:10.1007/s11042-018-6035-0

100. Kuang W, Ye W. A Kernel-Modified SVM Based Computer-Aided Diagnosis System in Initial Caries. 2008 Second International Symposium on Intelligent Information Technology Application. 2008;3: 207–211. Available: https://api.semanticscholar.org/CorpusID:18473867

101. Javed S, Zakirulla M, Baig RU, Asif SM, Meer AB. Development of artificial neural network model for prediction of post-streptococcus mutans in dental caries. Comput Methods Programs Biomed. 2020;186: 105198. doi:https://doi.org/10.1016/j.cmpb.2019.105198

102. Valizadeh S, Goodini M, Ehsani S, Mohseni H, Azimi F, Bakhshandeh H. Designing of a Computer Software for Detection of Approximal Caries in Posterior Teeth. Iranian Journal of Radiology. 2015;12. doi:10.5812/iranjradiol.12(2)2015.16242

103. Duong DL, Nguyen QDN, Tong MS, Vu MT, Lim JD, Kuo RF. Proof-of-Concept Study on an Automatic Computational System in Detecting and Classifying Occlusal Caries Lesions from Smartphone Color Images of Unrestored Extracted Teeth. Diagnostics. 2021;11. doi:10.3390/diagnostics11071136

104. Zheng L, Wang H, Mei L, Chen Q, Zhang Y, Zhang H. Artificial intelligence in digital cariology: a new tool for the diagnosis of deep caries and pulpitis using convolutional neural networks. Ann Transl Med. 2021;9: 763–763. doi:10.21037/atm-21-119

105. Oztekin F, Katar O, Sadak F, Yildirim M, Cakar H, Aydogan M, et al. An Explainable Deep Learning Model to Prediction Dental Caries Using Panoramic Radiograph Images. Diagnostics. 2023;13. doi:10.3390/diagnostics13020226

106. García-Cañas Á, Bonfanti-Gris M, Paraíso-Medina S, Martínez-Rus F, Pradíes G. Diagnosis of Interproximal Caries Lesions in Bitewing Radiographs Using a Deep Convolutional Neural Network-Based Software. Caries Res. 2022;56: 503–511. doi:10.1159/000527491

107. Pitts NB. Detection and measurement of approximal radiolucencies by computer-aided image analysis. Oral Surgery, Oral Medicine, Oral Pathology. 1984;58: 358–366. doi:10.1016/0030-4220(84)90068-9

108. Pitts NB, Renson CE. Reproducibility of Computer-aided Image-analysis-derived Estimates of the Depth and Area of Radiolucencies in Approximal Enamel. J Dent Res. 1985;64: 1221–1224. doi:10.1177/00220345850640100901

109. Pitts NB. Approximal radiolucencies in partially overlapped enamel: the need for quantitation and a preliminary assessment of a computer-aided image analysis method. Quintessence Int. 1986;17: 229–36.

110. Pitts NB, Renson CE. Further Development of a Computer-Aided Image Analysis Method of Quantifying Radiolucencies in Approximal Enamel. Caries Res. 2009;20: 361–370. doi:10.1159/000260959

111. Pitts NB. Detection of approximal radiolucencies in enamel: a preliminary comparison between experienced clinicians and an image analysis method. J Dent. 1987;15: 191–197. doi:10.1016/0300-5712(87)90108-4

112. Heaven TJ, Firestone AR, Feagin FF. Computer-based Image Analysis of Natural Approximal Caries on Radiographic Films. J Dent Res. 1992;71: 846–849. doi:10.1177/002203459207100S11

113. Heaven TJ, Weems RA, Firestone AR. The Use of a Computer-Based Image Analysis Program for the Diagnosis of Approximal Caries from Bitewing Radiographs. Caries Res. 1994;28: 55–58. doi:10.1159/000261621

114. DUNCAN RC, HEAVEN T, WEEMS RA, FIRESTONE AR, GREER DF, PATEL JR. Using Computers to Diagnose and Plan Treatment of Approval Caries Detected in Radiographs. The Journal of the American Dental Association. 1995;126: 873–882. doi:10.14219/jada.archive.1995.0308

115. Firestone AR, Sema D, Heaven TJ, Weems RA. The Effect of a Knowledge-based, Image Analysis and Clinical Decision Support System on Observer Performance in the Diagnosis of Approximal Caries from Radiographic Images. Caries Res. 1998;32: 127–134. doi:10.1159/000016442

116. Son LH, Tuan TM, Fujita H, Dey N, Ashour AS, Ngoc VTN, et al. Dental diagnosis from X-Ray images: An expert system based on fuzzy computing. Biomed Signal Process Control. 2018;39: 64–73. Available: https://api.semanticscholar.org/CorpusID:20089709

117. Ghaedi L, Gottlieb R, Sarrett DC, Ismail A, Belle A, Najarian K, et al. An automated dental caries detection and scoring system for optical images of tooth occlusal surface. 2014 36th Annual International Conference of the IEEE Engineering in Medicine and Biology Society. IEEE; 2014. pp. 1925–1928. doi:10.1109/EMBC.2014.6943988

118. Berdouses ED, Oulis CJ, Michalaki M, Tripoliti EE, Fotiadis DI. Histological validation of the automated caries detection system (ACDS) in classifying occlusal caries with the ICDAS II system in vitro. European Archives of Paediatric Dentistry. 2019;20: 249–255. doi:10.1007/s40368-018-0389-x

119. Rahman HA, Harun SW, Arof H, Irawati N, Musirin I, Ibrahim F, et al. Classification of reflected signals from cavitated tooth surfaces using an artificial intelligence technique incorporating a fiber optic displacement sensor. J Biomed Opt. 2014;19: 057009. doi:10.1117/1.JBO.19.5.057009

120. GAKENHEIMER DC. The efficacy of a computerized caries detector in intraoral digital radiography. The Journal of the American Dental Association. 2002;133: 883–890. doi:10.14219/jada.archive.2002.0303

121. Wenzel A, Hintze H, Kold LM, Kold S. Accuracy of computer‐automated caries detection in digital radiographs compared with human observers. Eur J Oral Sci. 2002;110: 199–203. doi:10.1034/j.1600-0447.2002.21245.x

122. Forner Navarro L, Llena Puy MC, García Godoy F. Diagnostic performance of radiovisiography in combination with a diagnosis assisting program versus conventional radiography and radiovisiography in basic mode and with magnification. Med Oral Patol Oral Cir Bucal. 2008;13: E261-5.

123. Moutselos K, Berdouses ED, Oulis CJ, Maglogiannis I. Superpixel-Based Classification of Occlusal Caries Photography. 2018 25th IEEE International Conference on Image Processing (ICIP). 2018; 1343–1347. Available: https://api.semanticscholar.org/CorpusID:52191142

124. Udod OA, Voronina HS, Ivchenkova OY. Application of neural network technologies in the dental caries forecast. Wiad Lek. 2020;73: 1499–1504.

125. Bhan A, Goyal A, Harsh, Chauhan N, Wang C-W. Feature Line Profile Based Automatic Detection of Dental Caries in Bitewing Radiography. 2016 International Conference on Micro-Electronics and Telecommunication Engineering (ICMETE). IEEE; 2016. pp. 635–640. doi:10.1109/ICMETE.2016.59

126. Naebi M, Saberi E, Risbaf Fakour S, Naebi A, Hosseini Tabatabaei S, Ansari Moghadam S, et al. Detection of Carious Lesions and Restorations Using Particle Swarm Optimization Algorithm. Int J Dent. 2016;2016: 1–7. doi:10.1155/2016/3264545

127. Datta S, Chaki N, Modak B. A Novel Technique to Detect Caries Lesion Using Isophote Concepts. IRBM. 2019;40: 174–182. doi:10.1016/j.irbm.2019.04.001

128. Al Kheraif AA, Wahba AA, Fouad H. Detection of dental diseases from radiographic 2d dental image using hybrid graph-cut technique and convolutional neural network. Measurement. 2019;146: 333–342. doi:10.1016/j.measurement.2019.06.014

129. Verma D, Puri S, Prabhu S, Smriti K. Anomaly detection in panoramic dental x-rays using a hybrid Deep Learning and Machine Learning approach. 2020 IEEE REGION 10 CONFERENCE (TENCON). IEEE; 2020. pp. 263–268. doi:10.1109/TENCON50793.2020.9293765

130. Jusman Y, Anam MK, Puspita S, Saleh E, Kanafiah SNAM, Tamarena RI. Comparison of Dental Caries Level Images Classification Performance using KNN and SVM Methods. 2021 IEEE International Conference on Signal and Image Processing Applications (ICSIPA). IEEE; 2021. pp. 167–172. doi:10.1109/ICSIPA52582.2021.9576774

131. Choudhary A, Raj G, Agrawal AP, Sawhney H, Nand P, Bhargava D. An Effective Approach for Classification of Dental Caries using Convolutional Neural Networks. 2021 10th International Conference on System Modeling & Advancement in Research Trends (SMART). IEEE; 2021. pp. 204–209. doi:10.1109/SMART52563.2021.9676250

132. Fariza A, Asmara R, Rojaby MOF, Astuti ER, Putra RH. Evaluation of Convolutional Neural Network for Automatic Caries Detection in Digital Radiograph Panoramic on Small Dataset. 2022 International Conference on Data and Software Engineering (ICoDSE). IEEE; 2022. pp. 65–70. doi:10.1109/ICoDSE56892.2022.9972183

133. Jusman Y, Widyaningrum A, Puspita S. Algorithm of Caries Level Image Classification Using Multilayer Perceptron Based Texture Features. 2022 IEEE International Conference on Cybernetics and Computational Intelligence (CyberneticsCom). IEEE; 2022. pp. 168–173. doi:10.1109/CyberneticsCom55287.2022.9865543

134. Jusman Y, Widyaningrum A, Tyassari W, Puspita S, Saleh E. Classification of Caries X-Ray Images using Multilayer Perceptron Models Based Shape Features. 2022 IEEE 7th International Conference on Information Technology and Digital Applications (ICITDA). IEEE; 2022. pp. 1–6. doi:10.1109/ICITDA55840.2022.9971452

135. Jayasinghe H, Pallepitiya N, Chandrasiri A, Heenkenda C, Vidhanaarachchi S, Kugathasan A, et al. Effectiveness of Using Radiology Images and Mask R-CNN for Stomatology. 2022 4th International Conference on Advancements in Computing (ICAC). IEEE; 2022. pp. 60–65. doi:10.1109/ICAC57685.2022.10025034

136. Liu F, Gao L, Wan J, Lyu Z-L, Huang Y-Y, Liu C, et al. Recognition of Digital Dental X-ray Images Using a Convolutional Neural Network. J Digit Imaging. 2022;36: 73–79. doi:10.1007/s10278-022-00694-9

137. Ramana Kumari A, Nagaraja Rao S, Ramana Reddy P. Design of hybrid dental caries segmentation and caries detection with meta-heuristic-based ResneXt-RNN. Biomed Signal Process Control. 2022;78: 103961. doi:10.1016/j.bspc.2022.103961

138. Dayı B, Üzen H, Çiçek İB, Duman ŞB. A Novel Deep Learning-Based Approach for Segmentation of Different Type Caries Lesions on Panoramic Radiographs. Diagnostics. 2023;13: 202. doi:10.3390/diagnostics13020202

139. Lin X, Hong D, Zhang D, Huang M, Yu H. Detecting Proximal Caries on Periapical Radiographs Using Convolutional Neural Networks with Different Training Strategies on Small Datasets. Diagnostics. 2022;12: 1047. doi:10.3390/diagnostics12051047

140. Majanga V, Viriri S. Automatic Blob Detection for Dental Caries. Applied Sciences. 2021;11: 9232. doi:10.3390/app11199232

141. Bui TH, Hamamoto K, Paing MP. Automated Caries Screening Using Ensemble Deep Learning on Panoramic Radiographs. Entropy. 2022;24: 1358. doi:10.3390/e24101358

142. Ari T, Sağlam H, Öksüzoğlu H, Kazan O, Bayrakdar İŞ, Duman SB, et al. Automatic Feature Segmentation in Dental Periapical Radiographs. Diagnostics. 2022;12: 3081. doi:10.3390/diagnostics12123081

143. Almalki YE, Din AI, Ramzan M, Irfan M, Aamir KM, Almalki A, et al. Deep Learning Models for Classification of Dental Diseases Using Orthopantomography X-ray OPG Images. Sensors. 2022;22: 7370. doi:10.3390/s22197370

144. Bui TH, Hamamoto K, Paing MP. Deep Fusion Feature Extraction for Caries Detection on Dental Panoramic Radiographs. Applied Sciences. 2021;11: 2005. doi:10.3390/app11052005

145. Chen Q, Huang J, Zhu H, Lian L, Wei K, Lai X. Automatic and visualized grading of dental caries using deep learning on panoramic radiographs. Multimed Tools Appl. 2023;82: 23709–23734. doi:10.1007/s11042-022-14089-z

146. Panyarak W, Wantanajittikul K, Charuakkra A, Prapayasatok S, Suttapak W. Enhancing Caries Detection in Bitewing Radiographs Using YOLOv7. J Digit Imaging. 2023;36: 2635–2647. doi:10.1007/s10278-023-00871-4

147. Suttapak W, Panyarak W, Jira-Apiwattana D, Wantanajittikul K. A unied convolution neural network for dental caries classication. ECTI TRANSACTIONS ON COMPUTER AND INFORMATION TECHNOLOGY. 2022;16. doi:10.37936/ecti-cit.2022162.245901

148. Ahmed WM, Azhari AA, Fawaz KA, Ahmed HM, Alsadah ZM, Majumdar A, et al. Artificial intelligence in the detection and classification of dental caries. J Prosthet Dent. 2023. doi:10.1016/j.prosdent.2023.07.013

149. Zadrożny Ł, Regulski P, Brus-Sawczuk K, Czajkowska M, Parkanyi L, Ganz S, et al. Artificial Intelligence Application in Assessment of Panoramic Radiographs. Diagnostics. 2022;12: 224. doi:10.3390/diagnostics12010224

150. Al-Jallad N, Ly-Mapes O, Hao P, Ruan J, Ramesh A, Luo J, et al. Artificial intelligence-powered smartphone application, AICaries, improves at-home dental caries screening in children: Moderated and unmoderated usability test. PLOS Digital Health. 2022;1: e0000046. doi:10.1371/journal.pdig.0000046
