# Supplementary figures and images for: Examining the diagnostic accuracy of artificial intelligence for detecting dental caries across a range of imaging modalities: An umbrella review with meta-analysis
Source: PLoS One. 2025 Aug 13;20(8):e0329986. doi: 10.1371/journal.pone.0329986 (PMC12349118; doi:10.1371/journal.pone.0329986)

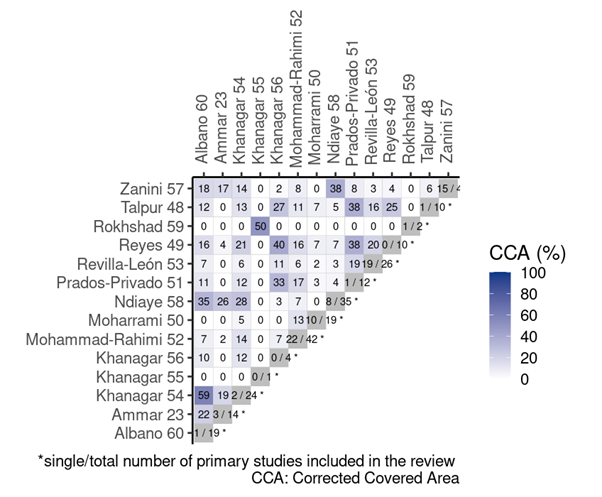

Supplement: S1 Fig — (TIF) [file pone.0329986.s002.tif]
