# Supplementary material for: Examining the diagnostic accuracy of artificial intelligence for detecting dental caries across a range of imaging modalities: An umbrella review with meta-analysis
Source: PLoS One. 2025 Aug 13;20(8):e0329986. doi: 10.1371/journal.pone.0329986 (PMC12349118; doi:10.1371/journal.pone.0329986)
Supplement: Attachment — Submitted filename: Rebuttal letter.docx [file pone.0329986.s004.docx]

**Editorial comments:**

In the case of your submission, we note that language restrictions are imposed on the literature searches without adequate justification. There is increasing recognition that exclusion of studies published in other languages can increase the risk of bias in systematic reviews. We feel that any restrictions should be justified based on scientific reasons rather than resource constraints. As a result, we have concerns about the comprehensiveness of the review and the validity of the conclusions.

**Response to editorial comments:**

As per the recommendation in the decision letter for PONE-D-25-03778, we have removed the English language restriction from our search strategy. A systematic search of all databases was updated, yielding no new results. The absence of additional findings aligns with the conclusions of Nussbaumer-Streit B et al. (2020) (<https://doi.org/10.1016/j.jclinepi.2019.10.011>), which stated that the exclusion of non-English publications from systematic reviews on clinical interventions has minimal impact on overall conclusions.
